# Expansion of induced pluripotent stem cells under consideration of bioengineering aspects: part 1

**DOI:** 10.1007/s00253-024-13372-3

**Published:** 2025-02-06

**Authors:** Samuel Lukas Schneider, Misha Alexander Teale, Stefan Seidel, Jürgen Krasenbrink, Martin Poggel, Dieter Eibl, Marcos F. Q. Sousa, Regine Eibl

**Affiliations:** 1 Centre for Cell Cultivation Techniques, Tissue Engineering, and Medical Biology, Institute of Chemistry and Biotechnology, ZHAW Zurich University of Applied Sciences, Grüentalstrasse 14, 8820 Wädenswil, Switzerland; 2 Advanced Manufacturing-Platform Engineering & Support, Bayer AG, Kaiser-Wilhelm-Allee 1, 51373 Leverkusen, Germany

**Keywords:** hiPSCs, Microcarriers, Perfusion, Scale-up, Single-use, Stirred bioreactor

## Abstract

**Abstract:**

To fully utilize the potential of human induced pluripotent stem cells (hiPSCs) for allogeneic stem cell–based therapies, efficient and scalable expansion procedures must be developed. For other adherent human cell types, the combination of microcarriers (MCs) and stirred tank bioreactors has been shown to meet these demands. In this study, a hiPSC quasi-perfusion expansion procedure based on MCs was developed at 100-mL scale in spinner flasks. Process development began by assessing various medium exchange strategies and MC coatings, indicating that the hiPSCs tolerated the gradual exchange of medium well when cultivated on Synthemax II–coated MCs. This procedure was therefore scaled-up to the 1.3-L Eppendorf BioBLU 1c stirred tank bioreactor by applying the lower limit of Zwietering’s suspension criterion (${N}_{s1u}$), thereby demonstrating proof-of-concept when used in combination with hiPSCs for the first time. To better understand the bioreactor and its bioengineering characteristics, computational fluid dynamics and bioengineering investigations were performed prior to hiPSC cultivation. In this manner, improved process understanding allowed an expansion factor of ≈ 26 to be achieved, yielding more than 3 × 10^9^ cells within 5 days. Further quality analyses confirmed that the hiPSCs maintained their viability, identity, and differentiation potential throughout cultivation.

**Key points:**

*•* ${N}_{s1u}$ *can be used as a scale-up criterion for hiPSC cultivations in MC-operated stirred bioreactors*

*• Uniform distribution and attachment of cells to the MCs are crucial for efficient expansion*

*• Perfusion is advantageous and supports the cultivation of hiPSCs*

**Supplementary Information:**

The online version contains supplementary material available at 10.1007/s00253-024-13372-3.

## Introduction

Human pluripotent stem cells hold immense potential for the development of novel cell therapies for the treatment of a wide variety of clinical indications (Flahou et al. 2021; Sivalingam et al. 2021; Eschenhagen et al. 2022). However, depending on the clinical indication, multiple doses containing anywhere between 10^5^ and 10^12^ cells are required if adequate treatment is to be guaranteed (Scibona and Morbidelli 2019). This is especially true for allogeneic cell therapy, where a single batch may be used to treat multiple patients (Pigeau et al. 2018; Lee et al. 2022), thereby placing additional demands on process yield if costs are to be kept within an acceptable range (Lee et al. 2020). One approach to addressing these demands is by scaling up existing cultivation processes (Pigeau et al. 2018). This, however, poses significant challenges for therapies based on hiPSCs, as the pluripotent nature and distinct phenotype of these cells make them particularly susceptible to fluid shear stress (Dang et al. 2021; Huang et al. 2021), uncontrolled aggregation (Kim et al. 2019; Huang et al. 2020; Dang et al. 2021), spontaneous differentiation (Leung et al. 2011), and depletion of substrate and growth factors in the cultivation medium (Horiguchi and Kino-oka 2021). Therefore, when developing manufacturing processes for therapies based on this cell type, the identification of bioengineering parameters that permit process scale-up while maintaining hiPSC growth and quality must be considered.

To date, various studies suggest cultivating hiPSCs as spheroids under stirred, serum-free conditions using bioengineering parameters such as the energy dissipation rate, shear stress, spheroid diameter, and tip speed as scale-up criteria (Huang et al. 2020; Dang et al. 2021; Borys et al. 2021; Cuesta-Gomez et al. 2023). However, given the challenges and low expansion factors (EF) of between 6 and 10 (Kwok et al. 2018; Kim et al. 2019; Huang et al. 2020) associated with these approaches, especially when transitioning to perfusion mode-operated L-scale stirred tank bioreactors (STBs), processes using MCs may yet demonstrate greater utility (Pandey et al. 2020).

In MC-operated cultivation processes, however, choosing a suitable stirring speed (N) is non-trivial. To characterize the suspension behavior of MCs in STBs, the ${N}_{s1}$ and ${N}_{s1u}$ suspension criteria have been developed. In this context, the ${N}_{s1}$ suspension criterion, as initially introduced by (Zwietering 1958), describes the N at which no particles are in contact with the bottom of the bioreactor for more than 1 s. The lower limit of this criterion, referred to as the ${N}_{s1u}$, further describes the N at which particles are in contact with the bottom of the cultivation vessel for extended periods of time, but are at rest for no longer than 1 s (Liepe et al. 1998; Wollny and Sperling 2021). Accordingly, the ${N}_{s1}$ criterion, which may also be found in literature under other names such as the “just suspended” [${N}_{js}$] (Ibrahim and Nienow 2004; Rafiq et al. 2017; Lawson et al. 2017) or “critical” stirring speed criterion [${N}_{c}$] (Petry and Salzig 2021), usually is 15–20% higher than ${N}_{s1u}$ (Kaiser et al. 2013; Jossen et al. 2014; Wollny and Sperling 2021).

For the cultivation of human mesenchymal stem cells (hMSCs), which place similar demands on MC-operated processes, both the ${N}_{s1}$ and ${N}_{s1u}$ have demonstrated practicality when scaling up beyond 35 L (Schirmaier et al. 2014; Lawson et al. 2017), especially when using geometrically dissimilar STBs with comparable fluid flow patterns and shear stress distributions (Schirmaier et al. 2014; Jossen et al. 2014). Given the increased sensitivity of hiPSCs to wall shear stress [τ] (Huang et al. 2021), the ${N}_{s1u}$ criterion should therefore be preferred over ${N}_{s1}$. Furthermore, since accurately identifying ${N}_{s1}$ and ${N}_{s1u}$ can be time-consuming and prone to investigator error, the development of computational fluid dynamics (CFD)-based approaches to support experimental characterization should also be pursued (Kaiser et al. 2013; Delafosse et al. 2018; Loubière et al. 2019).

**Fig. 1:**
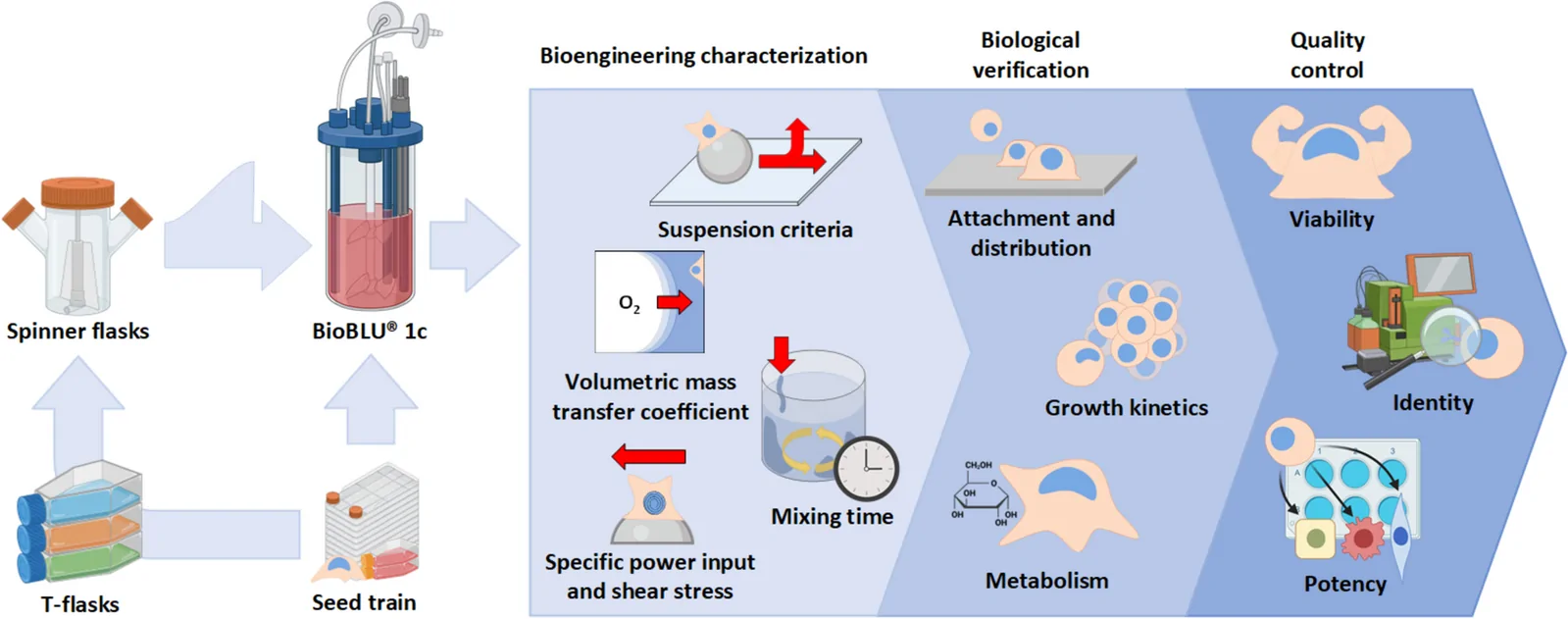
Illustration of the workflow used to accommodate the scale-up of a MC-operated, serum-free hiPSC expansion process to the SU BB1. Development began at mL-scale using T- and spinner flasks, after which the process was scaled up to the SU BB1 following the characterization of crucial bioengineering parameters. The ability of the ${N}_{s1u}$ criterion to support hiPSC expansion was subsequently confirmed through multiple L-scale experiments, during which cell attachment, distribution, growth, and metabolic activity were monitored. All biological experiments were assessed through daily sampling, while hiPSC quality was determined and compared by assessing viability, identity, and potency directly prior to inoculation and following harvest. Image partially created with Biorender.com

This article investigates for the first time whether > 10^9^ hiPSCs may be produced at L-scale using an MC-operated expansion process following scale-up between geometrically dissimilar single-use (SU) STBs using the ${N}_{s1u}$ criterion. As shown in Fig. 1, process development began with the characterization of the BioBLU 1c (BB1) alongside mL-scale experiments to determine optimal medium exchange (ME) regimes. The suitability of ${N}_{s1u}$ as a scale-up criterion between the fully characterized spinner flask (Kaiser et al. 2013; Jossen 2020) and BB1 was subsequently demonstrated through multiple L-scale experiments, accompanied by a comprehensive evaluation of hiPSC attachment, growth, metabolic activity, viability, identity, and differentiation potential.

## Materials and methods

### Bioengineering characterization of the BioBLU 1c

**Fig. 2:**
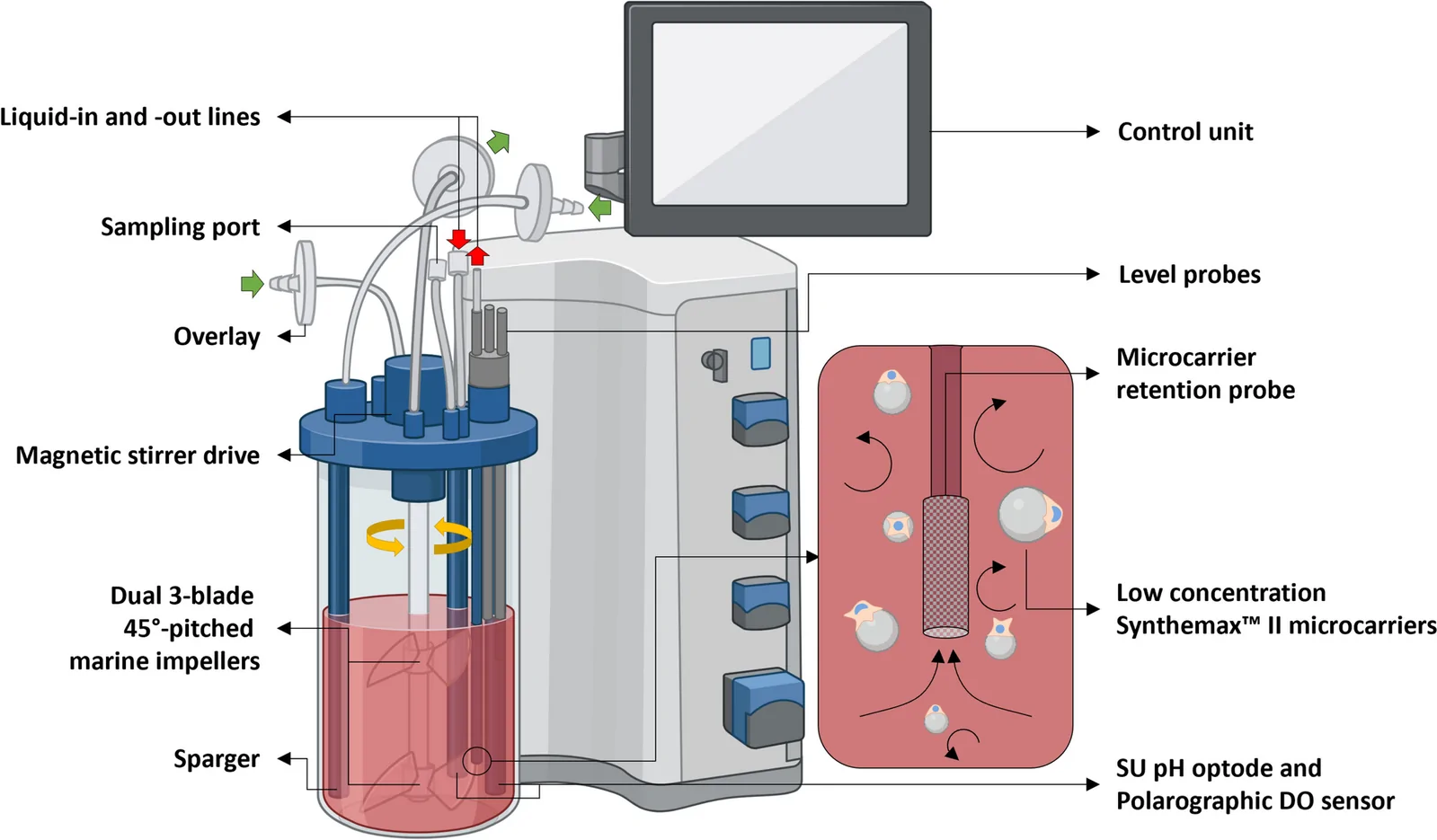
The SU BB1, used for the scale-up cultivations, is characterized by its flat bottom and features two 45°-pitched three-blade impellers spaced 70 mm apart. As clockwise and counter-clockwise stirring is possible (top view), the BB1 may be operated in either up- or down-pumping mode. Both impellers have a diameter (${d}_{R}$) of 50 mm, which, together with the maximum liquid height (${H}_{L}$) during operation of 160 mm, vessel diameter (${D}_{R}$) of 100 mm and impeller clearance to the base of 3.5 mm (${h}_{R}$), yield ${d}_{R}/{D}_{R}$, ${H}_{L}/{D}_{R}$, and ${h}_{R}/{D}_{R}$ ratios of 0.500, 1.600, and 0.035, respectively. Dissolved oxygen (DO) and pH control may be realized through a combination of headspace aeration and open-pipe sparging. At the same time, online monitoring of both values is facilitated through a built-in SU pH optode and a polarographic DO sensor port. During the experiments, the bioreactor was operated with either a BioFlo^®^ 320 bioprocess control system or with DASGIP Bioprocess Modules (Eppendorf AG, DE), with the growth surface provided by rigid, coated, non-porous, spherical MCs. Image partially created with Biorender.com

Similar to the modeling approach described by (Seidel et al. 2023b), the geometry of the BB1 (Fig. 2) (Eppendorf AG, DE) was drawn in Autodesk Inventor Professional 2023 (Autodesk Inc., US) prior to numerical characterization. Simulations with the model were then performed on a high-performance computing system using the open-source CFD toolbox OpenFOAM 10 (OpenFOAM Software, UK), after which Paraview 5.10 (Kitware Inc., US) and Python 3.10 (Python Software Foundation, US) were used for post-processing purposes as described in Seidel et al. (2023a).

Stationary single-phase simulations were carried out to evaluate the flow field and determine the specific power input and corresponding shear stress distribution (Wollny 2010), while Menter’s k-*ω* shear stress transport model was applied to model turbulence (Menter 1994). This turbulence model is particularly suitable for low Reynolds numbers, which are typical when cultivating stem cells. The interested reader will find a detailed derivation of the turbulence model in the publication by Seidel et al. (2023a). The stirrer rotation was modeled using the multiple reference frame approach, while no-slip boundary conditions were assumed for all surfaces. The physical properties of the modeled fluid corresponded to those of water at 37 °C and, since it may be assumed that no vortex formation would occur at the N of interest, the fluid surface was modeled with a symmetry plane.

The mixing time (${\theta }_{M}$) under various process conditions was determined transiently using a virtual tracer, whereby the steady-state flow field was used in this instance. For the ${N}_{s1u}$ suspension criterion, an Euler-Euler model was used, whereby the kinetic theory of granular flow (KTGF) was applied to simulate the MCs as a solid phase, similar to what has been described by Odeleye et al. (2020). For this simulation, a uniform MC diameter and density of 200 μm and 1025 kg m^−3^, respectively, was assumed. Theoretically, the densest possible packing uniform spheres allow for is 0.74, while more random packing has been shown to yield significantly lower densities of 0.60–0.64 (Wu et al. 2003). To account for more loose packing densities, which may still ultimately result in the formation of MC deposits as observed by Kaiser et al. (2013), a more conservative threshold of 0.37, corresponding to 50% of the maximum possible packing density, was selected for visualizing the spatial distribution of these deposits on the bioreactors base.

Alongside the numerical investigations, the suspension criteria ${N}_{s1}$ and ${N}_{s1u}$ were determined visually by filling a BB1 with 13 g of MCs with a diameter of 125–212 µm, a density of 1022–1030 kg m^−3^, and a surface area-to-mass ratio of 360 cm^2^ g^−1^ (Rafiq et al. 2016; García-Fernández et al. 2020). MC concentrations of 10.0–40.6 g L^−1^ were then achieved by adding phosphate-buffered saline (PBS) to a working volume (${V}_{L}$) of 0.3–1.3 L. To facilitate visual assessment, the bioreactor was suspended above an angled mirror to allow image capturing from the side. To further verify the results of the numerical models and establish an operating range, ${\theta }_{M}$, the volumetric mass transfer coefficient (${k}_{L}a$), and specific power input ($P/{V}_{L}$) were determined based on the recommendations made by DECHEMA (Bauer et al. 2020). Here, ${\theta }_{M}$ was assessed using the optical decolorization method, where the experiments were recorded by camera and the footage subsequently assessed to determine the timepoint of complete decolorization. The ${k}_{L}a$ was determined by applying the gassing-out method, and $P/{V}_{L}$ was measured via torque. The respective design space for these experiments encompassed a ${V}_{L}$ of 0.4–1.3 L and a N of 50–500 rpm, operated at a constant overlay gas flow rate (${F}_{c}$) of 195 mL min^−1^.

### Cell line and seed train preparation

All seed train expansions (2D-ST) were conducted using the commercially available Gibco™ Episomal TMOi001-A hiPSC line (Thermo Fisher Scientific Inc., US). Prior to the inoculation of the experiments, the hiPSCs were seeded at 1.0–4.5 × 10^4^ cells cm^−2^ on 0.5 µg cm^−2^ recombinant human vitronectin (rhVTN)–coated tissue culture (TC)–treated plates (Corning Inc., US) and expanded in either Essential 8™ Flex [E8F] (Thermo Fisher Scientific Inc., US) or mTesR1 [MR1] (STEMCELL Technologies, US) medium. The incubator was set to 37 °C, 5% CO₂, and 80% relative humidity. In all cultivations, the cells were seeded in medium supplemented with 10 µM Y-27632 (RI), a pan-rho-associated coiled-coiled kinase inhibitor (Miltenyi Biotec, DE). MEs were performed 24 h post-inoculation, then every 24–72 h, as recommended by the medium’s manufacturer. Before reaching a confluency of > 85%, the hiPSCs were either clump passaged using Versene (Thermo Fisher Scientific Inc., US) following a wash step with PBS or directly as single cells using Accutase^®^ (Corning Inc., US or STEMCELL Technologies, US) as described by Lai et al. (2022). Following detachment, the harvest reagent was quenched, the cell suspension spun down, the resulting supernatant discarded, and the cells resuspended in RI-supplemented culture medium in preparation for subsequent inoculation. In this manner, the cells were passaged at least twice before single-cell inoculation of the various cultivation systems.

### Process development at mL-scale

All mL-scale studies were conducted using E8F with a medium volume-to-surface area ratio (${V}_{L}/A$) of ≈ 0.28 mL cm^−2^. For the 2D cultivations, rhVTN-coated T25 flasks were seeded at 1.0 × 10^4^ cells cm^−2^ in RI-supplemented medium. All further MEs were conducted without RI supplementation and by applying one of three strategies. For the batch cultivations (2D-B), only a single 100% ME was performed 1 day after seeding to remove RI. For the repeated batch cultivations (2D-RB), 100% MEs were performed on days 1, 3, and 5. In comparison, for the quasi-perfusion cultivations (2D-QP), a 33% ME was conducted every 12 h starting 1 day post-inoculation, corresponding to a daily ME of 66% (185 μL cm^−2^ day^−1^). Cell growth and quality were evaluated by harvesting at least three flasks per ME strategy per day.

In preparation for the 3D cultivations, 125-mL disposable spinner flasks (Corning Inc., US) were each filled with 1 g of Low Concentration Synthemax^®^ II Microcarriers (Corning Inc., US) [SynII-PS], which display identical properties to those used to determine the suspension criteria, and 95 mL of RI-supplemented E8F, corresponding to a total growth surface of 360 cm^2^. The spinner flasks were subsequently inoculated with 2.0 × 10^4^ cells cm^−2^ and brought to a final ${V}_{L}$ of 100 mL, corresponding to a ${V}_{L}/A$ of ≈ 0.28 mL cm^−2^. Alongside appropriate coating selection (see Figure S1), cell attachment to the MCs was promoted by introducing a 12 h attachment phase. During this phase, N was set to 49 rpm, corresponding to ${N}_{s1u}$ for this vessel and MC combination (Kaiser et al. 2013), for 5 min to ensure homogenous cell suspension, after which stirring was stopped to permit cell attachment. Upon completion of the attachment phase, N was again set to ${N}_{s1u}$ for the remainder of the cultivation period. For the repeated batch cultivations (3D-RB), 100% MEs were conducted on days 2 and 4, while for the quasi-perfusion cultivation (3D-QP), 33% MEs were performed every 12 h (185 μL cm^−2^ day^−1^), starting 2 days after inoculation.

### Process scale-up using the ${{\boldsymbol{N}}}_{{\boldsymbol{s}}1{\boldsymbol{u}}}$ criterion

In total, four L-scale cultivations were performed using the BB1 bioreactors. As the processing method was continuously improved, the exact workflow differed slightly between each run (R). R1 and R2 were performed with E8F, while MR1 was used for R3 and R4. For all BB1 cultivations, preparations included filling the bioreactors with 0.3 L of RI-supplemented cultivation medium and 13 g of SynII-PS MCs (4 680 cm^2^) followed by the equilibration of the medium to a temperature, pH, and DO of 37 °C, 7.2, and 40%, respectively. For pH and DO, this was achieved solely through overlay aeration with N₂, O₂, CO₂, and air at a constant ${F}_{c}$ of 195 mL min^−1^.

Following equilibration, the bioreactors were inoculated with between 1.25 and 3.25 × 10^4^ cells cm^−2^ and brought to an initial ${V}_{L}$ of 0.4 L. Again, cell attachment and growth were promoted through intermittent stirring at 57 rpm (${N}_{s1u}$) at intervals of either 5 min every 175 min (R1 to 3) for 12 h or 5 min every 25 min for 6 h (R4), after which a constant N of 57 rpm was applied. To ensure homogeneous sampling, N was briefly increased to 85 rpm directly before and during sampling. After an initial 24 h batch phase, a 24 h fed-batch phase ensued, which added fresh medium to the bioreactor using a calibrated peristaltic pump set to ≈ 0.6 mL min^−1^ (≈ 185 μL cm^−2^ day^−1^). This brought the bioreactor to a final ${V}_{L}$ of 1.3 L and diluted RI to ≈ 3 µM or 33% of its original concentration. The fed-batch phase seamlessly transitioned into perfusion mode operation, with level probes intermittently activating a permeate pump when ${V}_{L}$ exceeded 1.3 L. During perfusion mode operation, the dilution rate (D) was increased daily in multiples of 0.66 day^−1^ to 1.32–1.98 day^−1^ (0.370–0.555 μL cm^−2^ day^−1^) according to the metabolic requirements of the cells, while MC retention was ensured using a custom built mesh covered dip tube (pore diameter ≈ 70–80 µm), similar to what has been described by Pandey et al. (2020) and Ullmann et al. (2024).

After 4–5 days, the bioreactors were sampled to allow for the calculation of harvest efficiency (HE). The bioreactors were then harvested at 20–25 °C by replacing the supernatant with 500 mL of harvest solution without a prior wash step. The harvest solution was based on a modified recipe by Jager et al. (2016) and consisted of TrypLE™ Select (Thermo Fisher Scientific Inc., US), DNase I (Merck, DE), and 10–20 µM RI. Following the addition of harvest solution, stirring was set to 57 rpm for 15 min, after which short intermittent pulses of up to 400 rpm were applied. Upon complete dissociation of the cells from the MCs, the harvesting solution was quenched with 500 mL of medium, after which the cells were separated from the MCs for further quality analyses using either 70 µm cell strainers (Corning Inc., US) or CliniMACS^®^ 600 mL Filtration Bags (Miltenyi Biotec, DE).

### Analytical techniques

#### Evaluation of cell count, distribution, confluency, and viability

Viable cell density (VCD) and viability were simultaneously evaluated following single cell harvest using either the NucleoCounter^®^ NC-200™ and Via1-cassettes™ or NC-202™ and Via2-cassettes™ (ChemoMetec, DK), respectively. Alongside the measurement of VCD, specific growth rates (μ), doubling times (${t}_{d}$), EF, attachment efficiencies (AE), cell distributions (CD), and also referred to as cell recovery rates, were quantified using established techniques described in greater detail elsewhere (Narumi et al. 2020; Teale et al. 2024).

#### Medium component analysis

Indirect monitoring of cell growth and death was achieved through daily bioreactor sampling and analysis of medium component concentrations or enzymatic activity within the supernatant. Sample analysis was conducted using the Cedex^®^ Bio Analyzer (Roche, CH) and corresponding reagent kits for glucose (Glc), glutamine (Gln), lactate (Lac), ammonium (NH4), and lactate dehydrogenase (LDH). When taken together with VCD, these analyses allowed for the calculation of cell-specific production and consumption rates (${q}_{s}$), alongside their respective yields (${Y}_{A/B}$), as reported by Teale et al. (2024).

#### Analysis of cell identity and potency

Before inoculation and following harvest, hiPSC quality was determined according to the techniques previously described by Teale et al. (2024). Briefly, the expression of the pluripotency markers Oct3/4, Sox2, Nanog, TRA-1–60, and SSEA-4, and the differentiation marker SSEA-1 was quantified for > 10^4^ cells using the MACSQuant^®^ 10 (Miltenyi Biotec, DE) flow cytometer (FCM) and suitable fluorophore-conjugated antibodies (Miltenyi Biotec, DE and BioLegend, US). Potency was assessed by plating the hiPSCs as single cells on rhVTN-coated TC-treated 6-well plates and bringing them to differentiate towards either an endo-, meso-, or ectodermal lineage over 5–7 days using the STEMdiff™ Trilineage Differentiation Kit (STEMCELL Technologies, CA). Successful differentiation was confirmed following single cell harvest, staining, and FCM analysis of > 10^4^ cells by quantifying marker combinations typical for either endo- (Sox17^+^ /CD184^+^), meso- (CD56^+^ /CD184^+^), and ectodermal (Nestin^+^ /Pax6^+^) tissue. Cells were treated with the Transcription Factor Staining Buffer Set (Miltenyi Biotec, DE) prior to staining all intracellular markers.

## Results

### Bioengineering characterization of the BioBLU 1c

**Fig. 3:**
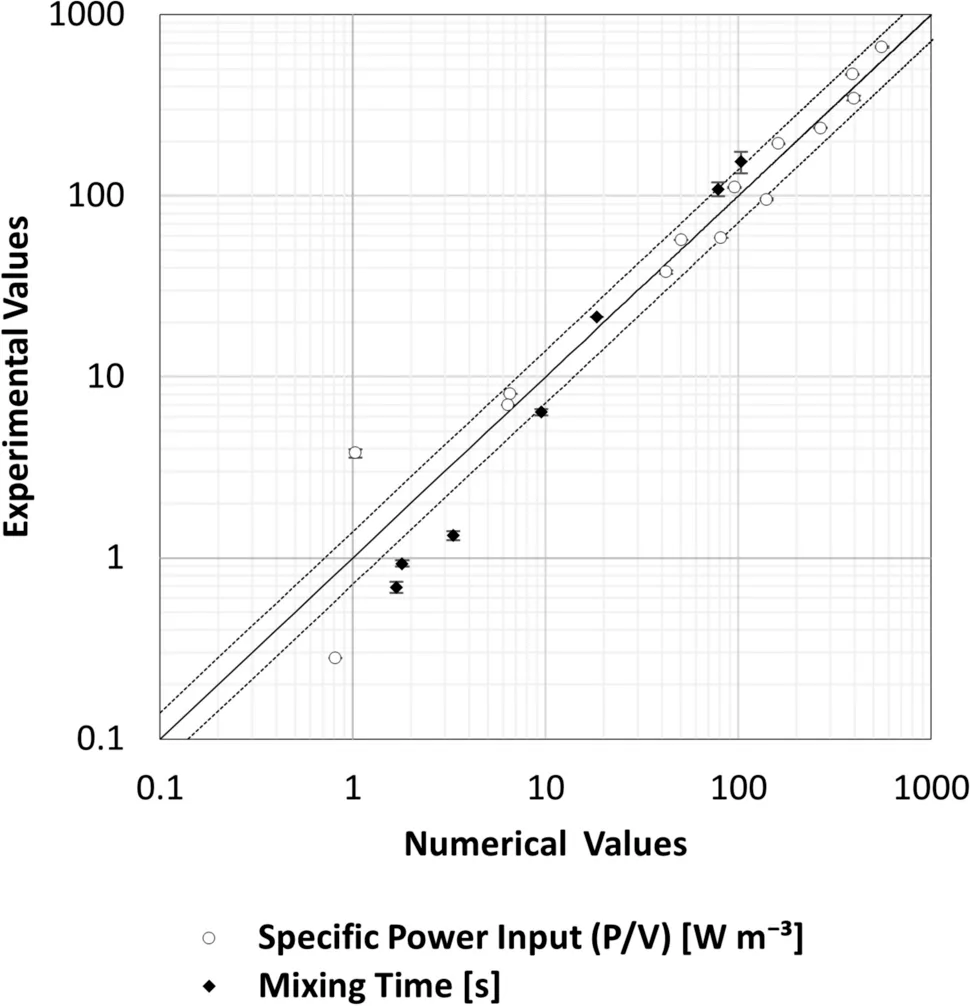
Comparison of numerical and experimental data values for $P/{V}_{L}$ and ${\theta }_{M}$ generated under identical process conditions. The solid identity line represents where values are equal, while the dotted lines illustrate the upper and lower 40% tolerance bands

To confirm the validity of the CFD simulations, a direct comparison was made between the numerically and experimentally determined values for $P/{V}_{L}$ and ${\theta }_{M}$ across various process conditions. As depicted in Fig. 3, such comparison indicated close agreement between methods beyond a critical threshold of 1 W m⁻^3^ or 4 s. Below these thresholds, technical limitations inherent to the experimental apparatus and methods, such as precise torque measurements and complementary mixing effects resulting from reagent addition, led to relative standard deviations exceeding 40%. Despite these challenges, the overall similarity between the numerical and experimental datasets when operating beyond the established thresholds demonstrated the integrity of these numerical models, justifying their use for predicting process conditions during operation in the lower range using CFD.

**Fig. 4:**
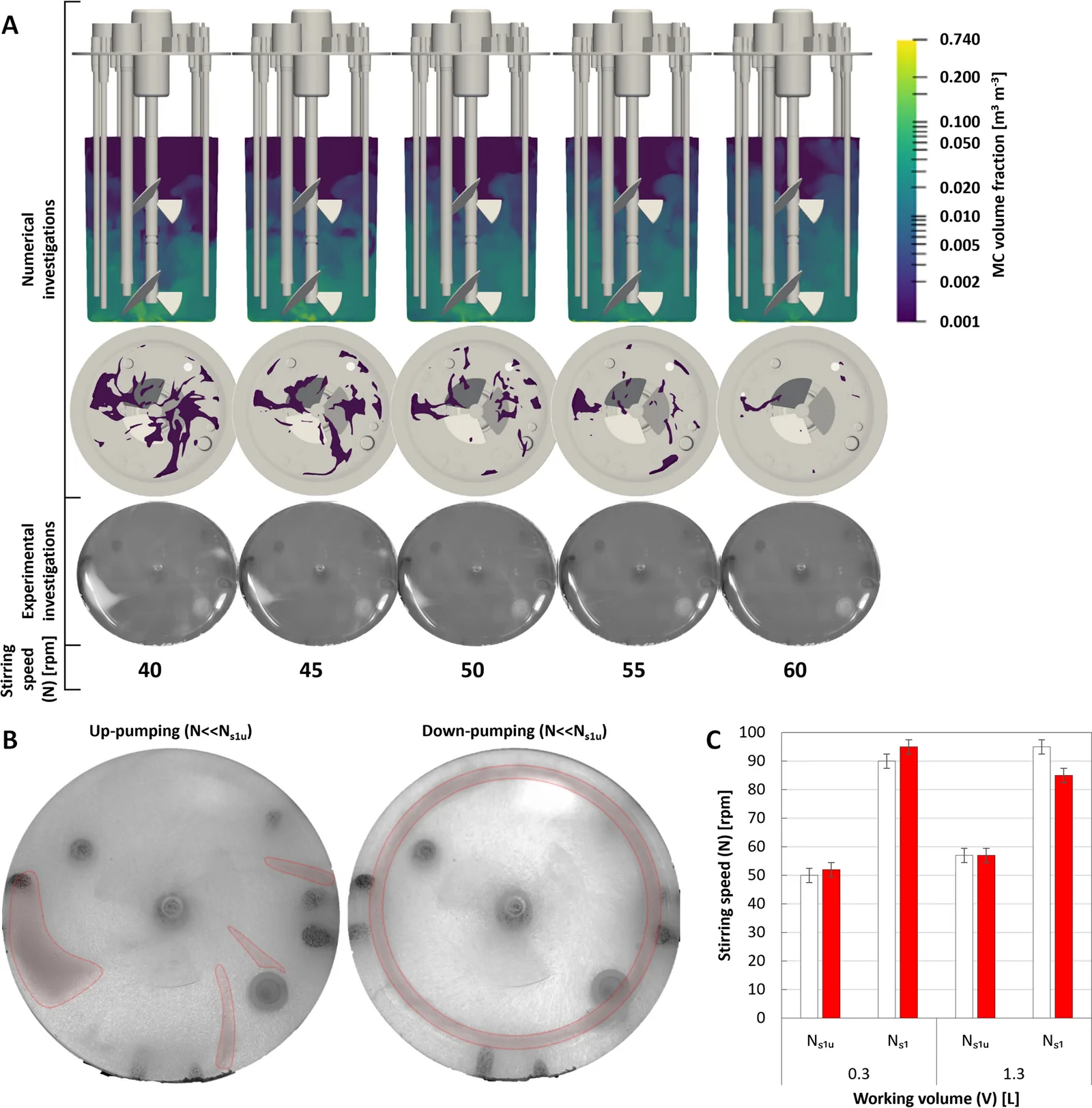
Numerical and experimental determination of the suspension criteria ${N}_{s1u}$ and ${N}_{s1}$ in the BB1. **A** Comparison of the numerically and experimentally observed MC sedimentation when operated in up-pumping mode between 40 and 60 rpm at a ${V}_{L}$ of 1.3 L when using 13 g of MCs. **B** SynII-PS MC sedimentation on the bottom of the BB1 (highlighted in red) when operated well below ${N}_{s1u}$ under up- and down-pumping conditions. **C** The experimentally determined N at which the ${N}_{s1u}$ and ${N}_{s1}$ criteria were met under (white) up-pumping or (red) down-pumping conditions when operated at either minimum or maximum ${V}_{L}$

Alongside the evaluation of $P/{V}_{L}$ and ${\theta }_{M}$, a more detailed assessment of ${N}_{s1u}$ was performed, given that this criterion served as the basis for scaling up the hiPSC expansion process from a spinner flask to the BB1. Initial predictions of MC behavior using the KTGF Euler-Euler model indicated the presence of MC concentration gradients following suspension (Fig. 4A). Consistent with the model, visual assessment during the experimental investigations confirmed the presence of these gradients and further facilitated the observation of MC deposition on the bioreactors base. More specifically, the size of these MC concentration gradients and deposits were shown to correlate inversely with N, diminishing as the inertia of the stationary MCs was overcome, and their more homogenous suspension throughout the liquid phase was ensured.

A more comprehensive comparison between the numerical and experimental datasets at 40–60 rpm indicated that these simulations could also predict the location and shape of the MC deposits when filtering for a critical volume fraction threshold of 0.37 or half the maximum packing density of these spheres. In both the numerical and experimental investigations, these localized MC deposits were found to form in the wakes of the internals when operated in up-pumping mode, especially at the tip of the harvest dip-tube, which had the lowest clearance to the base of the bioreactor. These deposits were further found to be dependent on the prevailing direction of fluid flow, as shown in the images captured of the BB1’s base when operated well below ${N}_{s1u}$ (Fig. 4B). Here, in contrast to the localized internal dependent deposits observed under up-pumping conditions, down-pumping operation allowed for a more dispersed, ring-shaped deposit to form at the base of the BB1’s wall, with the internals only marginally influencing its shape and distribution. Overall, the stirring speed required to reach the ${N}_{s1u}$ and ${N}_{s1}$ was only marginally influenced by the stirring direction. Nevertheless, as down-pumping mode offered a more homogeneous MC distribution, this stirring direction was selected for all further biological investigations. When operated in this manner and at a ${V}_{L}$ of 1.3 L, the ${N}_{s1u}$ criterion was met at 57 rpm, exposing the cells to a $P/{V}_{L}$ of 1.2 W m^−3^, a median shear stress ($\widetilde{\tau }$) of 0.27 × 10^−5^ N cm^−2^, and a 99th percentile shear stress (${\tau }_{99}$) of 5.46 × 10^−5^ N cm^−2^. Operation at this N was further shown to produce ${\theta }_{M}$ of 0.2–2.2 min and ${k}_{L}a$ of 1.0–3.8 h^−1^, when remaining within the minimum and maximum ${V}_{L}$ range.

#### Process development at mL-scale

**Fig. 5:**
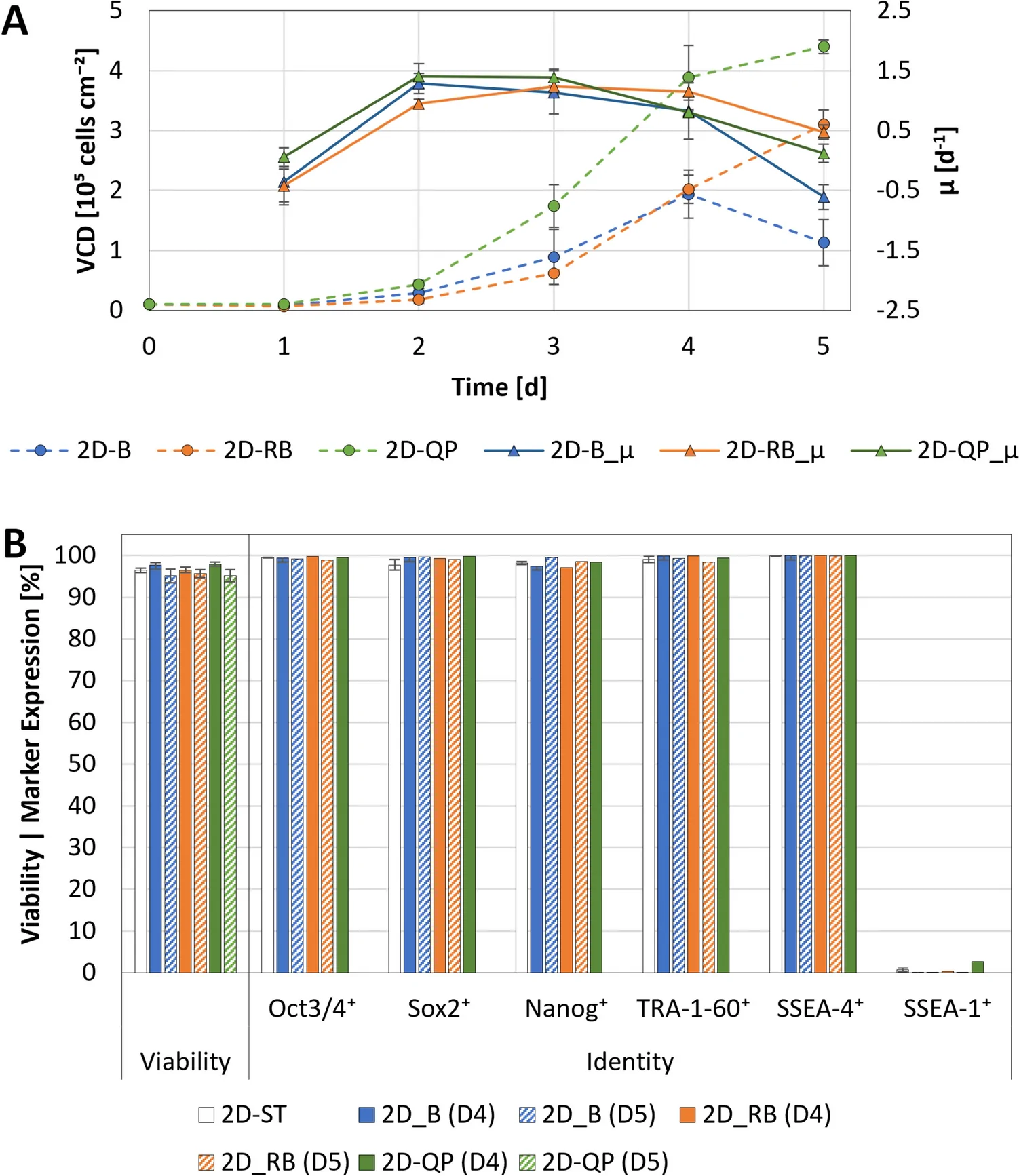
Growth and quality of hiPSCs cultivated in 2D under static conditions while applying different ME strategies. **A** Cell density and specific growth rate of the hiPSCs over the cultivation period. Each data point corresponds to an average of at least three replicates. **B** Quantification of cell viability and identity following harvest on days 4 and 5

Given the secondary aim of identifying an appropriate ME strategy, an initial step encompassed determining the impact of such a strategy on cell growth and quality at mL-scale. As shown in Fig. 5A, performing an ME 3 days post-inoculation on the 2D-RB replicates had no observable effect on cell density until day 4, compared to 2D-B. However, after day 4, pH in the 2D-B replicates dropped to 6.4 ± 0.0, leading to pH-mediated cell death. In contrast, alongside the gradual dilution of RI, the increased frequency of MEs in the 2D-QP experiments allowed pH to be kept above 6.5 ± 0.0 until day 5, resulting in an almost twofold higher VCD. This ME strategy also led to the highest recorded μ of 1.41 ± 0.16 day^−1^ or ${t}_{d}$ of 11.1 ± 1.3 h between days 1–2. While it is conceivable that the improved growth resulted from the applied ME strategy, minor differences in μ were already observed 1 day post-inoculation.

Depending on the ME strategy, the ideal time point for hiPSC harvest was shown to be 3.5–5 days post-inoculation, with the cells still in the late exponential phase and confluency not exceeding 85% (or ≈ 3.2 × 10^5^ cells cm^−2^). In all cases, harvesting the cells within this timeframe maintained their quality, as demonstrated through the quantification of viability and identity (Fig. 5B).

**Fig. 6:**
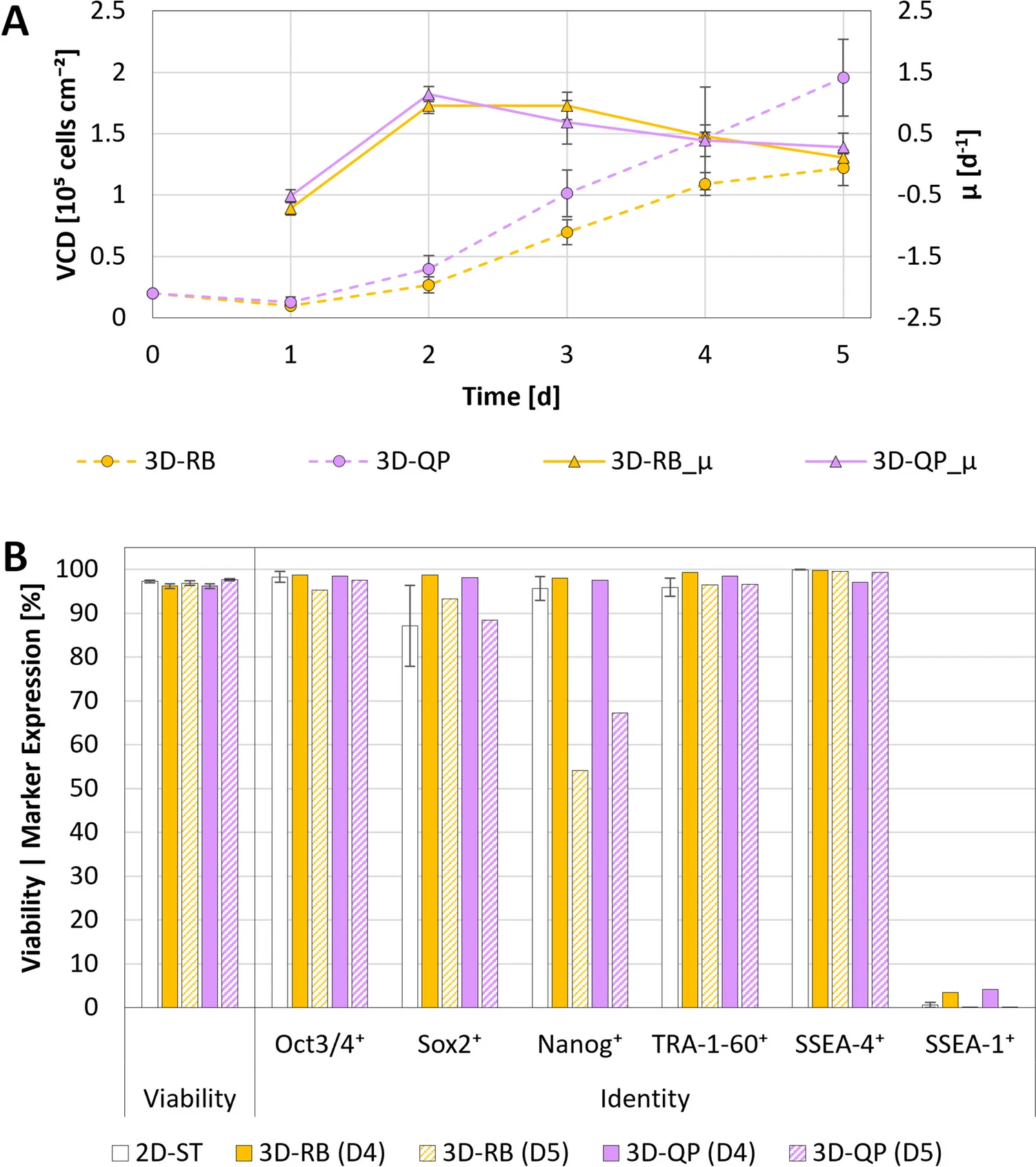
hiPSC growth and quality when cultivated in 3D using MC-operated spinner flasks and different ME strategies. **A** Cell density and specific growth rate of the hiPSCs over the cultivation period. Values correspond to a minimum of three single-cell harvests per data point. **B** Quantification of cell viability and identity following harvest on days 4 and 5.

Building on the results of the static experiments, the most promising ME strategies, 2D-RB and 2D-QP, were assessed for their suitability to support hiPSC growth in MC-operated spinner flasks at ${N}_{s1u}$. These experiments, conducted using commercially available SynII-PS MCs which had shown effectiveness in promoting hiPSC attachment and growth under static conditions (see supplementary data), resulted in twofold higher cell loss within 24 h post-inoculation, requiring the initial batch phase in RI-supplemented medium to be extended to support cell survival (Fig. 6A). While this prolonged exposure phase did not appear to have any adverse effects on growth or viability, with the highest μ of 1.15 ± 0.1 day^−1^ and ${t}_{d}$ of 14.6 ± 1.3 h observed between days 2 and 3, a notable decline in Sox2 and Nanog expression was observed between days 4 and 5 (Fig. 6B). Nevertheless, given that the quasi-perfusion approach again produced comparable results, a refined version of this ME strategy, where gradual RI dilution would occur between days 1 and 2, was selected for subsequent scale-up to the BB1.

#### Process scale-up to the BioBLU 1c using the ${{\boldsymbol{N}}}_{{\boldsymbol{s}}1{\boldsymbol{u}}}$ criterion

**Fig. 7:**
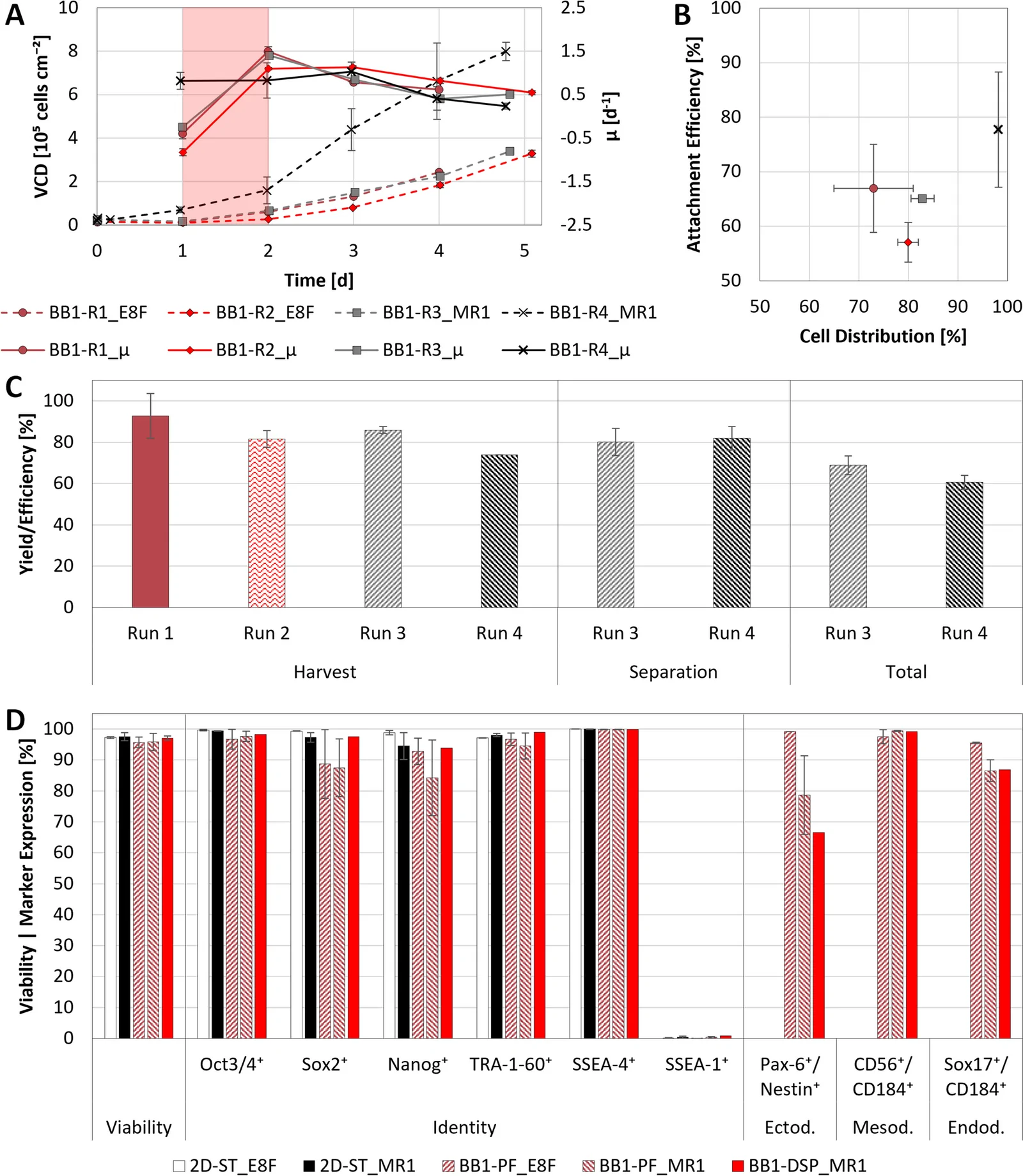
Growth, yield, and quality of hiPSCs expanded in MC-operated BB1s under serum-free conditions. **A** Viable cell density and specific growth rate during cultivation, consisting of an initial 24 h batch phase, followed by a 24 h fed-batch phase (red shading), and concluding with a seamless transition into perfusion mode operation. **B** AE and CD values of samples assessed 24 h following inoculation of the BB1s. **C** HE following cell harvest and separation. **D** Quantification of cell viability, identity, and differentiation potential following harvest and separation between days 4–5, grouped according to the serum-free medium used

Scaling up the process established in the spinner flasks to the BB1 under consideration of the ${N}_{s1u}$ criterion was characterized by a similar μ trend as observed during the mL-scale experiments, with a remarkable exception being the BB1-R4 (Fig. 7A). Here, a notably higher μ of 0.82 ± 0.19 day^−1^ was recorded between inoculation and day 1, alongside a twofold lower LDH activity of 47.5 U L^−1^. Given the minor differences between R3 and R4, this finding was attributed to changes made to the intermittent stirring regime, which increased AE and CD (Fig. 7B), lowering overall cell death.

After day 1, the hiPSC profile between R1–4 became comparable, with μ peaking between days 1 and 2 for R1–3, consistent with observations made during hiPSC cultivation at mL-scale. Contrary to the spinner flask cultivations, the combination of overlay aeration and perfusion mode enabled DO and pH to be maintained within defined ranges, with continuous medium replenishment particularly supporting stable pH control. As a result, pH remained between 6.8 and 7.2 throughout, while DO fell only briefly from its designated setpoint to ≈ 15% at the end of R4 as VCD exceeded 2.5 × 10^6^ cells mL⁻^1^. During this time, substrates and metabolites remained at non-limiting concentrations, with Glc and Gln above 8.3 mmol L⁻^1^ and 1.1 mmol L⁻^1^, and Lac and NH4 below 11.4 mmol L⁻^1^ and 1.5 mmol L⁻^1^, respectively.

Samples taken towards the end of the individual runs subsequently indicated that, when operated under these conditions, the MC-operated BB1s were able to produce between 1.5–3.3 × 10^9^ hiPSCs in a single 5-day run. When harvesting at these cell densities, brief pulses of N up to 400 rpm in R3 and R4 were shown to not have a noticeable impact on overall HE, compared to R1 and R2, which were exposed to a maximum N of 170 rpm. Rather, HE was shown to correlate inversely with the VCD at harvest (Fig. 7A, C), decreasing from 92.8 to 74.0% and suggesting that the presence of larger aggregates or hyperconfluence on the individual MCs could hinder enzymatic dissociation. Although unaffected by VCD, subsequent separation by dead-end filtration led to further cell loss, reducing the total cell yield to between 60 and 70% (Fig. 7C). Encouragingly, analyses performed on the harvested and filtered hiPSCs revealed no discernable impact of medium composition, harvest approach, or separation technique on cell quality, as substantiated through the quantification of viability, identity, and differentiation potential before and after each bioprocessing step (Fig. 7D).

## Discussion

Collectively, the reported findings suggest that when applied to the BB1, ${N}_{s1u}$ may be considered a suitable scale-up criterion for MC-operated hiPSC expansion processes, provided that both adequate cell attachment and distribution during inoculation are ensured. As shown in Table 1, from a technical perspective, this may be attributed to the bioengineering parameters, such as $P/{V}_{L}$, $\widetilde{\tau }$, ${\tau }_{99}$, ${\theta }_{M}$, and ${k}_{L}a$ aligning well with the ranges reported as acceptable for hiPSC expansion when operating the BB1 at ${N}_{s1u}$. Under these conditions, although twofold higher than reported for the spinner flasks (Jossen et al. 2018) at 1.2 W m^−3^, the numerically determined $P/{V}_{L}$ fell well within the optimal range of 0.3–1.5 W m^−3^ described for the expansion of hiPSCs as spheroids with similar diameters as the MCs in the Vertical Wheel (Dang et al. 2021; Cuesta-Gomez et al. 2023) and on MCs in the BioBLU 3c (Dorceus 2018; Pandey et al. 2020). Operation at ${N}_{s1u}$ also restricted hiPSC exposure to shear stress. Consequently, $\widetilde{\tau }$ and ${\tau }_{99}$ both remained below the 6.1 × 10^−5^ N cm^−2^ considered suitable for the cultivation of pluripotent stem cells in STBs (Cormier et al. 2006; Rohani et al. 2020), and well under the critical thresholds of 10 × 10^−5^ N cm^−2^, noted to elicit the upregulation of tissue-specific genes (Huang et al. 2021), and 100 × 10^−5^ N cm^−2^, known to trigger the detachment of adherently growing cells (Fuhrmann and Engler 2015).

**Table 1:** Comparison of hiPSC growth and quality during process development using various SU bioreactors operated in quasi-perfusion or perfusion mode. Values for the bioengineering parameters of the spinner flasks were adapted from Jossen (2020)

Parameter	T25-Flask	Spinner Flask	BioBLU^®^ 1c dual-impeller	
${V}_{L}/A$ [mL cm^−2^]	0.28	0.28	0.28	
${N}_{s1u}$ [rpm]	n.a	49	57	
$P/{V}_{L}$ [W m^−3^]	n.a	0.63	1.20	
$\widetilde{\tau }$ [× 10^−5^ N cm^−2^]	n.a	n.d	0.27	
${\tau }_{99}$ [× 10^−5^ N cm^−2^]	n.a	n.d	5.46	
${k}_{L}a$ [h^−1^]	n.d	1.3	1.0–3.8	
${\theta }_{M}$ [min]	n.d	0.2 ± 0.0	0.4–2.2	
Operation mode	Quasi-perfusion	Quasi-perfusion	Perfusion	
Medium	E8F	E8F	E8F	MR1
Coating	rhVTN	SynII	SynII	SynII
Attachment phase [h]	24	12	12	6–12
${q}_{Glc}$ [pmol cell^−1^ day^−1^]	13.4 ± 7.9	18.5 ± 6.7	18.0 ± 5.7	6.1 ± 2.4
${q}_{Gln}$ [pmol cell^−1^ day^−1^]	2.7 ± 2.1	2.6 ± 1.1	2.7 ± 1.0	0.9 ± 0.4
${q}_{Lac}$ [pmol cell^−1^ day^−1^]	22.5 ± 10.8	33.8 ± 11.4	30.7 ± 9.1	10.7 ± 5.7
${q}_{NH4}$ [pmol cell^−1^ day^−1^]	1.6 ± 0.9	1.9 ± 0.7	1.8 ± 0.7	0.6 ± 0.3
${q}_{O_{2}}$ [pmol cell^−1^ h^−1^]	n.d	n.d	0.443 ± 0.162	0.292 ± 0.082
${Y}_{Lac/Glc}$ [mol mol^−1^]	1.8 ± 0.2	1.8 ± 0.2	1.7 ± 0.2	1.7 ± 0.2
${Y}_{NH4/Gln}$ [mol mol^−1^]	0.9 ± 0.2	0.8 ± 0.1	0.7 ± 0.1	0.7 ± 0.2
Max. viable cell yield [cells]	9.7 ± 1.3 × 10^6^	5.3 ± 1.0 × 10^7^	1.7 ± 0.1 × 10^9^	3.3 ± 0.2 × 10^9^
Max. EF [-]	38.9 ± 5.3	7.3 ± 1.3	21.5 ± 1.0	25.5 ± 1.3
Cultivation time [day]	4	4	4–5	5
Min. ${t}_{d}$ [h]	11.1 ± 1.3	14.6 ± 1.3	11.1 ± 0.4	11.9 ± 0.4
Max. HE [%]	n.d	n.d	92.8 ± 10.9	85.9 ± 1.7
Viability [%]	98.0 ± 0.5	98.0 ± 0.5	95.7 ± 1.9	95.8 ± 3.0
Pluripotent	Yes	Yes	Yes	Yes
Tri-lineage differentiation	n.d	n.d	Yes	Yes

*n.a* not applicable, *n.d* not determined

Alongside $P/{V}_{L}$ and τ, effective cell expansion in MC-operated STBs also necessitates efficient attachment and homogenous distribution of cells on the MCs during the post-inoculation attachment phase (Derakhti et al. 2019; Teale et al. 2024). This is especially true for hiPSCs, given their characteristically poor motility (Zhang et al. 2011), the discontinuous nature of the growth surface provided by MCs (Tsai and Pacak 2021), and the challenges associated with bead-to-bead transfer (Badenes et al. 2017). This was best exemplified by the almost twofold higher AE of 106.1 ± 9.3% observed for the 2D-QP cultivations compared to replicates cultivated in 3D, resulting in a ≈ 25% lower ${t}_{d}$ between days 1 and 2 and a more than fivefold higher EF at the end of cultivation. This issue was, therefore, addressed during the BB1 cultivations by optimizing the intermittent stirring regime during the attachment phase. The applied changes, which encompassed increasing intermittent frequency and shortening the duration of the attachment phase in R4, improved both AE and CD from between 57.0 and 66.9% and 73.0–82.9% to 77.7 ± 10.6% and 98.2 ± 0.3% following the first 24 h, respectively. These adjustments led to a positive μ after day 1, a comparable μ to R1–3 between days 1 and 2, and an almost twofold higher yield of ≈ 3.3 × 10^9^ cells by day 5.

Meeting the intrinsic metabolic needs of the hiPSCs at such densities meant maintaining sufficient mixing and an adequate supply of oxygen alongside the continuous replacement of essential medium components during cultivation. Here, a comparison of values reported for spinner flasks (Jossen 2020) and other L-scale SU bioreactors, such as the BioBLU^®^ 3c (Dorceus 2018; Pandey et al. 2020), Mobius^®^ 3 L (Kwok et al. 2018; Kreitmayer et al. 2022b), Xcellerex™ XDR-10 (Huang et al. 2020; Kreitmayer et al. 2022a), and Ascent™ 1 m^2^ (Teale et al. 2025), suggest that hiPSC densities of more than 6 × 10^6^ cells mL^−1^ are achievable without loss of quality when operating at ${\theta }_{M}$ and ${k}_{L}a$ of between 0.1–1.5 min and 0.4–10 h^−1^, respectively. Given that operating the BB1 at ${N}_{s1u}$ exclusively with overlay gassing resulted in similar ${\theta }_{M}$ and ${k}_{L}a$ of between 0.4–2.2 min and 1.0–3.8 h^−1^, the bioreactor may be considered suitable for the cultivation of hiPSCs at similar densities. This was subsequently demonstrated during R4, where a VCD of ≈ 2.6 × 10^6^ cells mL^−1^ was achieved by day 5, during which time DO remained within a suitable operating range of 15–40% (Abecasis et al. 2017; Horiguchi and Kino-oka 2021). Nevertheless, it should be mentioned that, while not ideal for hiPSC cultivation (Nogueira et al. 2021), the inclusion of sparging would undoubtedly increase ${k}_{L}a$ even further (Dashtban et al. 2021).

By taking into account VCD together with ${k}_{L}a$ and the prevailing DO gradient, the ${q}_{{O}_{2}}$, as described by Abecasis et al. (2017), could be calculated between each sampling point, allowing further insight into the hiPSC metabolic tendencies. Here, remarkable differences in ${q}_{{O}_{2}}$ between days 2 and 5 were noted depending on the serum-free medium used (Table 1). More specifically, a linear decrease in the range of 0.443 ± 0.162 pmol cell^−1^ h^−1^ was observed for the E8F experiments, while ${q}_{{O}_{2}}$ in the MR1 experiments increased, remaining within the range of 0.292 ± 0.082 pmol cell^−1^ h^−1^ over the same period. Given that the hiPSCs reported ${q}_{{O}_{2}}$ of between 0.01 and 4.38 pmol cell^−1^ h^−1^ (Abecasis et al. 2017; Greuel et al. 2019; Teale et al. 2025) is dependent on the prevailing DO conditions (Abecasis et al. 2017), the cell’s pluripotent state (Teslaa and Teitell 2015), and substrate availability (Horiguchi and Kino-oka 2021), the values and trends observed during the BB1 experiments were considered plausible.

Alongside ${q}_{{O}_{2}}$, medium composition was also shown to impact Glc and Gln metabolism (Table 1). Although similar to the values reported by Manstein et al. (2021), when cultivated in E8F, the cells displayed two- to threefold higher uptake and production rates than when cultivated in MR1. A subsequent evaluation of the respective yields, however, revealed that the ratio of aerobic glycolysis to oxidative phosphorylation or ${Y}_{Lac/Glc}$ remained comparable, irrespective of cultivation vessel and medium used, aligning well with the values reported in literature (Kropp et al. 2016; Teale et al. 2024, 2025). This also held true for ${Y}_{NH4/Gln}$ (Teale et al. 2024). In all cultivations, ${Y}_{Lac/Glc}$ briefly increased to ≈ 2.0 following the dilution of RI, followed by a steady decline towards ≈ 1.6, suggesting an increase in oxidative phosphorylation. For the T-flask and BB1 experiments, this occurred on day 2, while during the spinner flask experiments, where RI was removed or diluted 24 h later, this was observed to happen on day 3. Given that RI is a known mesoendodermal differentiation primer (Maldonado et al. 2016), this impact on cell metabolism was not unexpected. Thus, the implementation of a fed-batch and perfusion phase during the BB1 cultivations not only prevented limitations resulting from Glc, Gln, Lac, and NH4 (Chen et al. 2010; Horiguchi et al. 2018) but likewise facilitated the timely removal of RI, minimizing its impact on cell metabolism.

Alongside the improvements made to the attachment phase, it could further be shown that perfusion mode operation facilitated the production of almost 3 × 10^6^ cells mL^−1^ within 5 days at an EF of 25.5 ± 1.3. Such cell densities are comparable with the > 2 × 10^6^ cells mL^−1^ produced by Pandey et al. (2020) within 5–9 days when using the MC-operated BioBLU 3c. Similar to what was observed for the kSep^®^ Centrifuge (Pandey et al. 2020) and Sefia™ Cell Processing System (Huang et al. 2020), dead-end filtration was shown to reduce yield by ≈ 20%, without significant impact to hiPSC viability, identity, or differentiation potential. Following separation, viabilities remained > 95.7%, with > 84.3% of the cell population expressing all pluripotency markers and > 78.7% differentiating into all three germ layers within 5–7 days.

In closing, assuming that a dose corresponds to ≈ 10^9^ cells and that the hiPSCs have been adequately differentiated, a single BB1 produced enough cells to treat up to three patients. Therefore, considering that the largest commercially available BioBLU^®^ bioreactor, namely the BioBLU^®^ 50c, has a ${V}_{L}$ of 40 L, subsequent scale-up of the reported process using the ${N}_{s1u}$ criterion could potentially facilitate the production of more than ≈ 100 doses per batch. Whether other SU bioreactors, such as those reported for viral vector and extracellular vesicle production with adherently growing mammalian cell lines, may also be used for this purpose is subsequently investigated in the second part of this two-part publication (Teale et al. 2025).

## Supplementary Information

Below is the link to the electronic supplementary material.

Supplementary file1 (PDF 254 KB)

## Data Availability

The datasets generated and/or analyzed during the current study and the code used, are available from the corresponding author upon reasonable request.

## Abbreviations

2D-B: Static cultivation conducted in batch mode
2D-QP: Static cultivation conducted in quasi-perfusion mode
2D-RB: Static cultivation conducted in repeated batch mode
2D-ST: Seed train conducted under static conditions
3D-QP: Dynamic cultivation conducted in quasi-perfusion mode
3D-RB: Dynamic cultivation conducted in repeated batch mode
BB1: Dual-impeller BioBLU^®^ 1c bioreactor
CFD: Computational fluid dynamics
DO: Dissolved oxygen
E8F: Essential 8™ Flex
FCM: Flow cytometry
Glc: Glucose
Gln: Glutamine
hiPSC: Human induced pluripotent stem cell
hMSC: Human mesenchymal stem cell
KTGF: Kinetic theory of granular flow
Lac: Lactate
LDH: Lactate dehydrogenase
MC: Microcarrier
ME: Medium exchange
MR1: mTeSR™ 1
NH4: Ammonium
PBS: Phosphate-buffered saline
R: Run
rhVTN: Recombinant human vitronectin
RI: Pan rho-associated, coiled-coil protein kinase inhibitor Y-27632
STB: Stirred tank bioreactor
SU: Single-use
SynII-PS: Low Concentration Synthemax^®^ II Microcarriers
TC: Tissue culture

## Latin symbols

AE: -, Attachment efficiency
CD: -, Cell distribution
D: s^−^^1^, Dilution rate
$${d}_{R}$$: m, Impeller diameter
$${D}_{R}$$: m, Vessel diameter
${d}_{R}/{D}_{R}$: -, Impeller-to-tank diameter ratio
EF: -, Expansion factor
${F}_{c}$: m^3^ s^−^^1^, Combined gas flow rate
$${h}_{R}$$: m, Impeller clearance
${H}_{L}$: m, Maximum liquid height in the bioreactor
${h}_{R}/{D}_{R}$: -, Impeller clearance-to-tank diameter ratio
${H}_{L}/{D}_{R}$: -, Bioreactor aspect ratio
HE: -, Harvest efficiency
${k}_{L}a$: s^−^^1^, Volumetric mass transfer coefficient
N: s^−^^1^, Stirring speed
${N}_{c}$: s^−^^1^, The “critical” stirring speed criterion
${N}_{js}$: s^−^^1^, The “just stirred” criterion
${N}_{s1}$: s^−^^1^, Zwieterings suspension criterion
${N}_{s1u}$: s^−^^1^, The lower limit of Zwieterings suspension criterion
$P/{V}_{L}$: kg m^−^^1^ s^−^^3^, Specific power input
${q}_{s}$: mol cell^−^^1^ s^−^^1^, Cell-specific consumption/production rate of compound s
$${t}_{d}$$: s, Doubling time
$${V}_{L}$$: m^3^, Working volume
${V}_{L}/A$: m, Working volume-to-surface area ratio
VCD: cells m^−^^3^, Viable cell density
${Y}_{A/B}$: mol mol^−^^1^, Yield of A from B

## Greek symbols

$${\theta }_{M}$$: s, Mixing time
μ: s^−^^1^, Specific growth rate
τ: N m^−^^2^, Wall shear stress
$\widetilde{\tau }$: N m^−^^2^, Median wall shear stress
${\tau }_{99}$: N m^−^^2^, 99^th^ percentile wall shear stress
