# Supplementary material for: Expansion of induced pluripotent stem cells under consideration of bioengineering aspects: part 1
Source: Appl Microbiol Biotechnol. 2025 Feb 6;109:37. doi: 10.1007/s00253-024-13372-3 (PMC11802619; doi:10.1007/s00253-024-13372-3)
Supplement: Supplementary file 1 [file 253_2024_13372_MOESM1_ESM.pdf]

Expansion of induced pluripotent stem cells under consideration of bioengineering aspects: Part 1 – Supplementary data

Samuel Lukas Schneider [†1], *Misha Alexander Teale* [1†], *Stefan Seidel* [1], *Jürgen Krasenbrink* [2], *Martin Poggel* [2], *Dieter Eibl* [1], *Marcos F. Q. Sousa* [2*], *Regine Eibl* [1]

[a] Centre for Biochemical Engineering and Cell Cultivation Techniques, Institute of Chemistry and Biotechnology, Zurich University of Applied Sciences, Grüentalstrasse 14, 8820 Wädenswil, Switzerland. samuel.schneider@zhaw.ch https://orcid.org/0000-0002-2756-408X (S.L.S.), misha.teale@zhaw.ch, https://orcid.org/0000-0003-0599-4115 (M.A.T.), stefan.seidel@zhaw.ch, https://orcid.org/0000-0002-5244-0855, (S.S.), dieter.eibl@zhaw.ch, https://orcid.org/0000-0003-0033-3393 (D.E.), regine.eibl@zhaw.ch, https://orcid.org/0000-0002-1840-8253 (R.E.)

[b] Advanced Manufacturing-Platform Engineering & Support, Bayer AG, Kaiser-Wilhelm-Allee 1, 51373 Leverkusen, Germany. juergen.krasenbrink@bayer.com (J.K.), marcos.sousa1@bayer.com, https://orcid.org/0000-0003-3766-3900 (M.F.Q.S.), martin.poggel@bayer.com (M.P.)

[*] Corresponding author

[†] These authors contributed equally to the writing of this manuscript.

SUPPLEMENTARY DATA

Scaffold coating study

If hiPSCs do not attach to a cell-adhesion mediators (CAMs)-coated scaffold, they either form spheroids through cell-cell adhesion or undergo dissociation-induced apoptosis (Kim et al. 2019). Therefore, given the importance of scaffold selection, three different MCs were chosen for screening under static conditions at mL-scale, namely Enhanced Attachment Microcarriers [EA-PS] (Corning Inc., US), Low Concentration Synthemax II Microcarriers [SynII-PS] (Corning Inc., US), and SynII coated Dissolvable Microcarriers [SynII-DS] (Corning Inc., US). All MCs were purchased pre-sterilized and, where applicable, pre-coated. To further promote hiPSC attachment, the surface of the EA-PS MCs was additionally coated with 0.5 µg cm^{-2} of rhVTN (EArhVTN-PS). Uniform coating was achieved by placing the MCs in 50 mL centrifuge tubes (Corning Inc., US) and suspending them for 1 h at 25 °C in rhVTN-supplemented PBS using an incubator shaker (Infors AG, CH) set to 200 rpm (25 mm shaking diameter). Once coated, the EArhVTN-PS MCs were allowed to sediment, the supernatant discarded, and the MCs resuspended to a concentration of 10 g L^{-1} in fresh RI-supplemented E8F in preparation for the screening experiments.

The screening experiments were conducted by transferring the equivalent of 7.2 cm^2 of each MC type suspended in 2 mL of E8F supplemented RI to every well of multiple ultra-low attachment (ULA) 6-well plates (3–6 replicates per MC type). For these experiments, uncoated EA-PS MCs acted as negative controls. The MCs were then inoculated with single cells (1.0–2.0 × 10^4 cells cm^{-2}), briefly mixed to ensure uniform distribution of the hiPSCs and MCs, and subsequently placed in a static incubator set to 37 °C, 5 % CO_2, and 80 % relative humidity (rH) for 24 h to promote cell attachment. After 24 h, a 90 %

ME was performed with fresh E8F without removing any MCs. This step was followed by an additional 48 h of static incubation at 37 °C, 5 % CO_2, and 80 % rH.

Single-cell harvest of the adherently growing hiPSCs was initiated 72 h post-inoculation by removing 90 % of the spent medium in each well and using 1 mL of TrypLE™ Select (Thermo Fisher Scientific Inc., US) to transfer the MCs 2 mL centrifuge tubes (Eppendorf AG, DE). These centrifuge tubes were then orbitally shaken at 37 °C, 1000 rpm for 5 min using a ThermoMixer® C (Eppendorf AG, DE) to facilitate cell detachment. For the SynII-DS MCs, the proteolytic reagent was further supplemented with Versene and Pectinase (Merck, DE) to allow the MC scaffold to be dissolved during harvest. Once detached, cell viability and EF were compared, and statistical analyses conducted using Prism 10 (Graphpad, US). Significance was determined using a one-way analysis of variance followed by Tukey's honest significance post-test. Differences were considered statistically significant if $p < 0.05$ (*).

The results of the screening study (see Figure S1) clearly emphasize the importance of CAMs when ensuring adequate hiPSC attachment to and growth on the various MCs. This was best demonstrated by directly comparing the coated (EArhVTN-PS, SynII-PS, SynII-DS) and uncoated (EA-PS) MC types. While the cells harvested from the uncoated EA-PS MCs were characterized by a similar viability of 90.2 ± 6.7 % compared to those cultivated on the coated MCs, overall cell growth was significantly lower with an EF of 0.6 ± 0.6 after 3 d. On the contrary, the hiPSCs harvested from the coated EArhVTN-PS, SynII-PS, and SynII-DS MCs were characterized by respective viabilities of 93.7 ± 1.4, 89.9 ± 2.3, and 91.1 ± 2.3, achieving significantly higher EF of 3.8 ± 1.0, 3.9 ± 1.0, and 3.6 ± 0.4. Remarkably, no differences between the MCs based on their coating or the polymer composition of their scaffold could be discerned. Therefore, given the outcome

of the screening study, the rigid nature of the PS scaffold, and the potency of the SynII CAM, the SynII-PS MCs were selected for further investigation under dynamic conditions at mL- and L-scale.

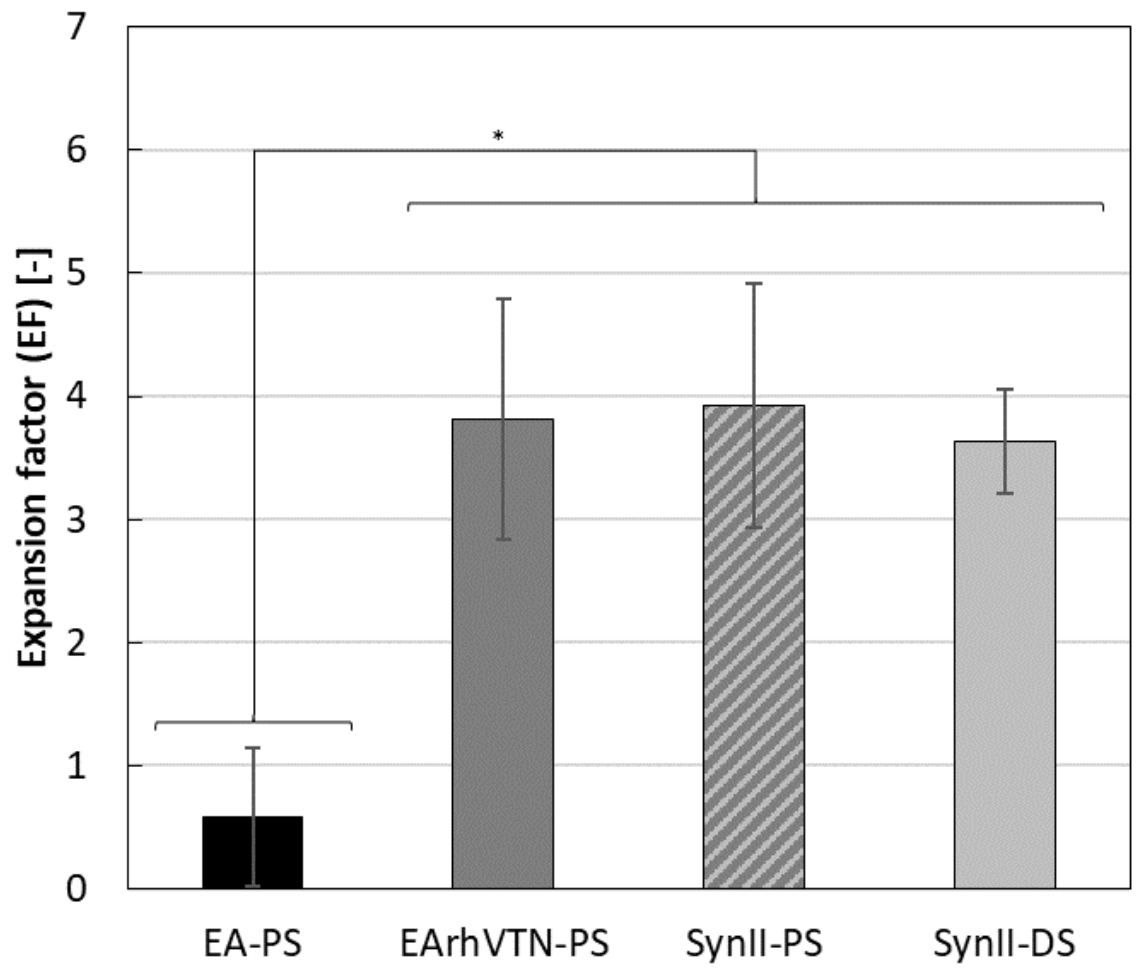

Figure S1: Evaluation of various MC types to support cell attachment and growth. CAM- and scaffold-mediated hiPSC attachment and growth on the various MC types were assessed by quantifying cell count following 3 d of cultivation. * $p < 0.05$.
